# Temporal Changes in Local Functional Connectivity Density Reflect the Temporal Variability of the Amplitude of Low Frequency Fluctuations in Gray Matter

**DOI:** 10.1371/journal.pone.0154407

**Published:** 2016-04-26

**Authors:** D. Tomasi, E. Shokri-Kojori, N. D. Volkow

**Affiliations:** 1 National Institute on Alcohol Abuse and Alcoholism, Bethesda, Maryland, United States of America; 2 National Institute on Drug Abuse, Bethesda, Maryland, United States of America; University Of Cambridge, UNITED KINGDOM

## Abstract

Data-driven functional connectivity density (FCD) mapping is being increasingly utilized to assess brain connectomics at rest in the healthy brain and its disruption in neuropsychiatric diseases with the underlying assumption that the spatiotemporal hub distribution is stationary. However, recent studies show that functional connectivity is highly dynamic. Here we study the temporal variability of the local FCD (*l*FCD) at high spatiotemporal resolution (2-mm isotropic; 0.72s) using a sliding-window approach and ‘resting-state’ datasets from 40 healthy subjects collected under the Human Connectome Project. Prominent functional connectivity hubs in visual and posterior parietal cortices had pronounced temporal changes in local FCD. These dynamic patterns in the strength of the *l*FCD hubs occurred in cortical gray matter with high sensitivity (up to 85%) and specificity (> 85%) and showed high reproducibility (up to 72%) across sessions and high test-retest reliability (ICC(3,1) > 0.5). The temporal changes in *l*FCD predominantly occurred in medial occipitoparietal regions and were proportional to the strength of the connectivity hubs. The temporal variability of the *l*FCD was associated with the amplitude of the low frequency fluctuations (ALFF). Pure randomness did not account for the probability distribution of *l*FCD. Shannon entropy increased in proportion to the strength of the *l*FCD hubs suggesting high average flow of information per unit of time in the *l*FCD hubs, particularly in medial occipitoparietal regions. Thus, the higher dynamic range of the *l*FCD hubs is consistent with their role in the complex orchestration of interacting brain networks.

## Introduction

The complex and time-varying operations of the human brain require a dynamic brain network topology to support the context-dependent coordination of neural populations [1]. However, most studies on ‘resting-state’ functional connectivity (FC) assume that brain networks are stationary [2] and rely on short data acquisition [3]. Since this dynamic behavior enhances the within-subject variability of the functional connectivity metrics [4], accounting for the dynamic changes in connectivity could facilitate the development of FC biomarkers for clinical applications in neurology and psychiatry [2] and could also reveal dynamic properties of brain topology [5]. In addition, development of voxelwise approaches that capture dynamic FC changes could help identify brain regions that are prone to high within-subject variability in FC studies.

Functional magnetic resonance imaging (fMRI) studies on FC are based on image time series collected at rest [6], in the presence of temporal changes in the degree of vigilance, memory, arousal and attention. During data acquisition, over periods of several minutes, the human brain sequentially engages in a series of diverse free-streaming subject-driven cognitive states supported by different brain networks [7–10], and previous studies have investigated the origins of the time-varying FC signals [11–13]. Dynamic connectivity patterns have been observed using the sliding-window approach with seed-voxel correlation or with independent component (ICA) analyses. These studies have identified significant temporal variability in lateral parietal and cingulate cortices and in the default-mode network [5,14,15]. However, the temporal variability of the local functional connectivity density (*l*FCD), a graph theory metric, has not been quantified.

Graph theory functional connectivity density mapping (FCDM) quantifies local degree, the size of the local network cluster functionally connected to a brain network node, and is a powerful voxelwise data-driven tool for exploring the topology of the human brain connectome [16]. In contrast to seed-voxel correlation analysis [6], data driven FCDM is ideal for exploratory analyses because it quantifies the strength of the local functional connectivity hubs (network nodes with high connectivity to nearby brain regions), in just a few minutes/subject [16] and does not rely on a priory hypotheses. These characteristics make FCDM optimal for data mining in large resting-state FC repositories [3,17]. We and others have shown the predominance of *l*FCD hubs in posterior parietal and occipital cortices [16,18] that are influenced by age [19] and gender [20], stimulant drugs [21], fluid reasoning capacity [22], brain development [23] and dopamine signaling [24]. FCDM is being increasingly utilized to assess brain function at rest in neuropsychiatric populations. For instance, *l*FCD hubs are disrupted in attention deficit hyperactivity disorder [17], cocaine addiction [21], non-epileptic seizures [25], schizophrenia [23,26,27], congenital blindness [28] and traumatic axonal injury [29].

Whereas FC promises to have a major impact in neuroscience, its significant within-subject variability might limit its potential as a clinical biomarker for neuropsychiatric diseases. We hypothesized that temporal dynamics account for a significant fraction of the within-subject variability in *l*FCD patterns.

Here we capitalize on datasets with unprecedented spatiotemporal resolution (2-mm isotropic; 0.72 s) recently released by the Human Connectome Project (HCP; https://db.humanconnectome.org/)[30], which offer a unique opportunity for mapping the temporal variability of the *l*FCD hubs in the human brain with high spatial specificity [31]. Inasmuch as the amplitude of the spontaneous signal fluctuations reflect the integrated coordination of neuronal activity [32] we hypothesized that the temporal variability in the strength of the *l*FCD, as measured by the standard deviation of the *l*FCD as a function of time, would be proportional to the temporal variability in the amplitude of the spontaneous signal fluctuations in the low frequency band (0.01–0.08Hz). We also predicted that the temporal variability in the *l*FCD would be proportional to the magnitude of the FC.

We mapped the temporal evolution of the *l*FCD over a 14-min time interval using a sliding-window approach and resting-state FC datasets from 40 healthy adults acquired under the HCP. We quantified gray matter sensitivity and specificity and reproducibility across sessions of the variability of the *l*FCD as a function of time, while controlling for global effects by using global signal normalization (GSN). We also assessed the effect of different thresholds in the computation of the *l*FCD. We hypothesized that *l*FCD hubs in posterior parietal and occipital gray matter would show the largest temporal variability in *l*FCD. To test the dynamic linear association between the *l*FCD and the amplitude of the signal fluctuations as a function of time in each individual we mapped the amplitude of the low frequency fluctuations (ALFF) in the brain [33].

## Methods

### Subjects

Data were drawn from the publicly available repository of the WU-Minn HCP (http://www.humanconnectome.org/). No experimental activity with any involvement of human subjects took place at the author's institutions. The 40 participants (age: 31 ± 3 years; 31 females) of the WU-Minn HCP Q1 data release included in this study provided written informed consent and were scanned on a 3.0T Siemens Skyra unit equipped with a 32-channel radiofrequency head coil according to procedures approved by the IRB at Washington University in St. Louis.

### Datasets

Resting-state functional images were acquired while the participant relaxed with eyes open using a gradient-echo-planar sequence with multiband factor 8, TR 720 ms, TE 33.1 ms, flip angle 52 deg, 104 × 90 matrix size, 72 slices, 2 mm isotropic voxels, and 1200 timepoints [34,35]. Scans were repeated twice using different phase encoding directions (left-right, LR, and right-left, RL) in each of the two imaging sessions (REST1 and REST2). The “minimal preprocessing” datasets, which include gradient distortion correction, rigid-body realignment, field-map processing, spatial normalization to the stereotactic space of the Montreal Neurological Institute (MNI), high pass filtering (1/2000 Hz frequency cutoff) [36], independent component analysis-based denoising [37], and brain masking were used in this study. One hundred and sixty ‘resting-state’ time series (2 sessions × 2 phase encoding directions × 40 subjects) with 1200 time points (864s) and 2mm-isotropic voxels (whole brain coverage) were used in this study. In addition we used the HCP’s gray and white matter parcellations of each subject’s brain structural scans, to create a gray matter template. This template was used to assess the gray matter specificity of the *l*FCD. The interactive data language (IDL, ITT Visual Information Solutions, Boulder, CO) and a workstation with two Intel^®^ Xeon^®^ X5680 processors were used in subsequent processing steps to compute dynamic *l*FCD and ALFF brain maps, for each subject, session (REST1, REST2) and phase encoding direction (LR, RL).

### Local degree

The *l*FCD at every voxel in the brain, *x*_0_, was computed as the number of voxels in the contiguous functional connectivity cluster of *x*_0_ using a "growing" algorithm [16]. The Pearson correlation was used to assess the strength of the functional connectivity, *R*_*ij*_, between voxels *i* and *j* in the brain, and a correlation threshold *R*_*ij*_ > 0.4, was selected to ensure significant correlations between time-varying signal fluctuations at P < 0.0001, uncorrected. A voxel (*x*_*j*_) was added to the list of voxels functionally connected with *x*_0_ only if it was adjacent to a voxel that was linked to *x*_0_ by a continuous path of functionally connected voxels and *R*_0*j*_ > 0.4. This calculation was repeated for all brain voxels that were adjacent to those that belonged to the list of voxel functionally connected to *x*_0_ in an iterative manner until no new voxels could be added to the list.

### Amplitude of fluctuations

The preprocessed time series were also used to map ALFFfor all voxels in the brain. Specifically, the fast Fourier transform was used to compute the ALFF as the average of the power spectrum's square root in the 0.01–0.08 Hz low frequency bandwidth [33]. The Pearson linear correlation was used to map for each individual time series the association between dynamic FC metrics (ALFF and *l*FCD) as a function of time.

### Sliding-window

To quantify the time-varying behavior of *l*FCD and ALFF over the duration of the scan, we used a fixed rectangular time window with N = 100 image time points (72s). Data points within the time window were used to calculate *l*FCD and ALFF patterns. The time window was subsequently shifted by N/2 (36s) and the *l*FCD and ALFF patterns were then recalculated. This was repeated 23 times to cover the entire scanning window. A fixed rectangular time window with N = 200 image time points (144s) was used to assess the effect of window length. In addition Hamming windows,

$$h(n)=0.5\left(1+\text{cos}\left(\frac{2\pi n}{N-1}\right)\right),$$ (1)

of lengths N = 100 (72 sec) and N = 200 (144 sec) time points were used to assess the effect of sliding-window shape.

### Dynamic motion

Framewise displacements, FD, were computed for every time point from head translations (*d*_*ix*_, *d*_*iy*_, *d*_*iz*_) and rotations (*α*_*i*_, *β*_*i*_, *γ*_*i*_) using a radius of *r* = 50 mm:

$$FD_{i}=\left|\Delta d_{ix}\right|+\left|\Delta d_{iy}\right|+\left|\Delta d_{iz}\right|+r\left|\Delta \alpha_{i}\right|+r\left|\Delta \beta_{i}\right|+r\left|\Delta \gamma_{i}\right|.$$ (2)

In order to assess the influence of head motion on the dynamic *l*FCD patterns the sliding-window approach was used to quantify the average FD within each sliding-window frame. Specifically, correlation analyses were used to assess the linear association between dynamic FD and *l*FCD or ALFF. These Pearson correlation maps from each subject were then normalized using the Fisher’s transformation and used to test the association between the temporal variability of head motion and those of *l*FCD or ALFF.

### Temporal variability

The temporal standard deviation (SD) of the time varying *l*FCD patterns was used to quantify the variability of this measure as a function of time at each brain voxel.

### Data processing pipelines

**Fig 1:**
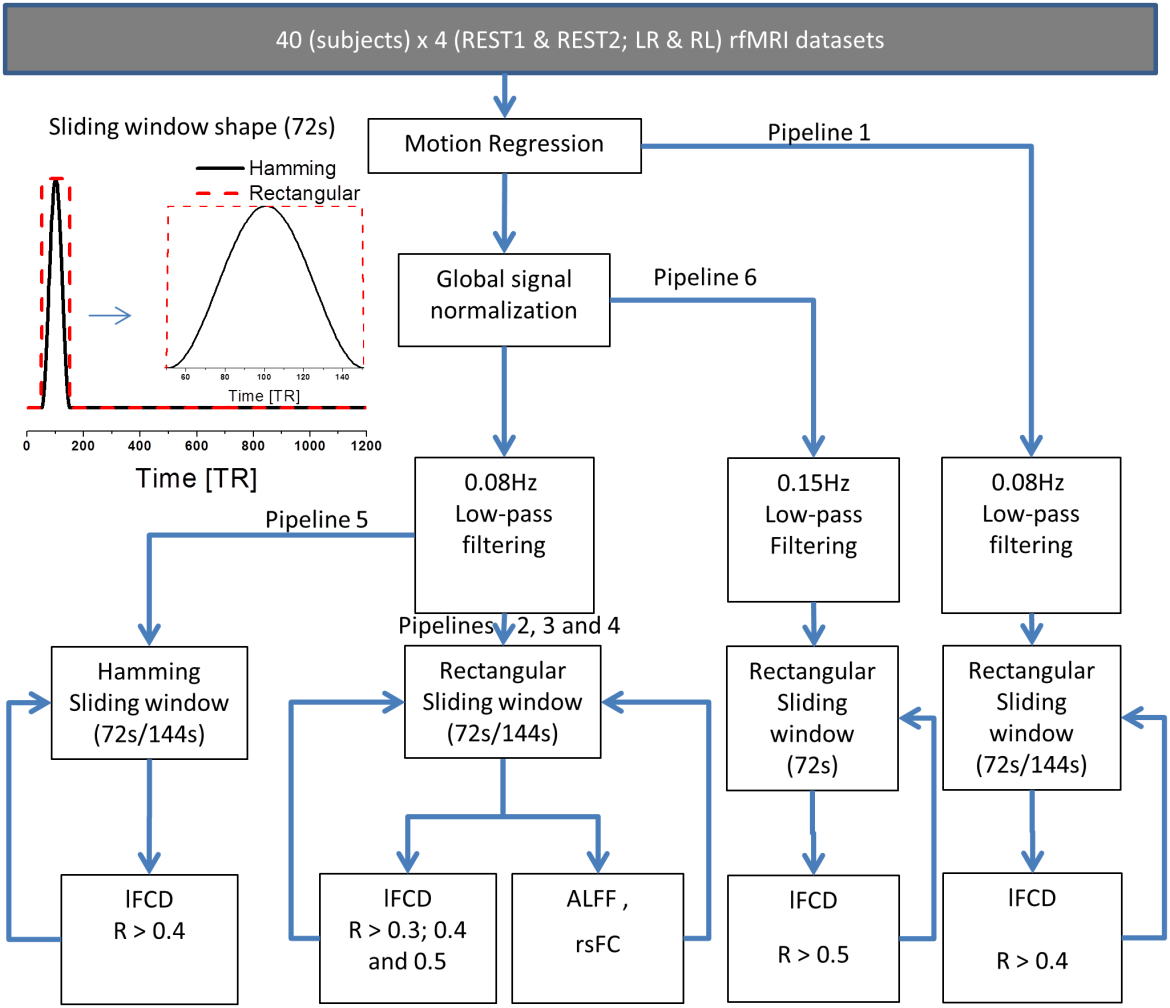
Image processing pipelines. Ten dynamic *l*FCD maps and 4 dynamic ALFF maps were computed for each subject, session, and phase encoding direction using 5 different pipelines (see text). A total of 1600 *l*FCD and 640 ALFF maps covering the whole brain (white matter and cerebrospinal fluid regions were not masked out to assess the strength of the *l*FCD in these regions) with 2-mm isotropic resolution and 91×109×91 voxels were computed using 160 HCP datasets with “minimal preprocessing” [36] from the Q1 release. Smoothing was not used to preserve the high spatial resolution of the resting-state functional datasets.

Six pipelines were implemented (Fig 1). Pipeline 1 included a multilinear regression approach to minimize motion related fluctuations in the MRI signals [16]. 3D GSN was performed to minimize global fluctuations and to account for linear and non-linear drifts in the MRI signal:

$$S\left(x,y,z,t\right)=S\left(x,y,z,t\right)\times 1000\times M\;/\sum_{x,y,z}S\left(x,y,z,t\right),$$ (3)

where *M* is the number of voxels within a brain mask and *S*(*x*, *y*, *z*, *t*) is the MRI signal from a voxel at time *t*. Standard 0.08 Hz low-pass filtering was used to minimize physiologic noise of high frequency components. The strengths of ALFF and *l*FCD maps were computed as described using the sliding-window approach. Then the SD maps were computed as described. Spatial smoothing was not used in order to preserve the high spatial resolution of the native datasets. The *l*FCD was evaluated in the whole brain using the whole brain mask (227372 ± 2461 voxels; mean ± standard deviation). Five alternative pipelines avoiding GSN with *R*_0*j*_ > 0.3 (pipeline 2); *R*_0*j*_ > 0.4 (pipeline 3); *R*_0*j*_ > 0.5 (pipeline 4); using Hamming sliding-windows (pipeline 5) or with a 0.15Hz low-pass filter (pipeline 6) were additionally implemented to assess the effects of global signal fluctuations, correlation thresholds, sliding-window shape and low-pass filtering parameters on the temporal variability of the *l*FCD at high spatiotemporal resolution.

### Grand mean global scaling

The mean SD in the whole brain of each individual was averaged across subjects and voxels

$$\langle SD\rangle =\frac{1}{LM}\sum_{k=1}^{S}\sum_{i=1\;}^{L}{SD}_{i}^{k},$$ (4)

where *L* is the number of subjects and *M* is the number of voxels in the brain, and used as the unique scaling factor, 1/<SD>, for a given pipeline. This scaling procedure allowed us to control for global differences associated with differences in thresholds, sliding-window shapes and GSN conditions in statistical analyses. Similar grand mean global scaling was used for the *l*FCD.

### Sensitivity, specificity and reproducibility indices

We used three indices to benchmark the effect of global signal regression and correlation thresholds on the temporal variability of the *l*FCD [31]:

$$Sensitivity=\frac{M_{GM}}{M_{tissue}}\frac{\sum_{i\in \{tissue\}}SD_{i}}{\sum_{i\in \{brain\}}SD_{i}},$$ (5)

which gauges the proportion of SD within the tissue of interest (i.e. cortical or subcortical gray matter or white matter), normalized to gray matter volume (M_tissue_/M_GM_);

$$Specificity=\frac{\sum_{i\in \{\text{WM}\}}\varepsilon }{\sum_{i\in \{\text{WM}\}}1};\;\varepsilon =\{\begin{array}{ll} 1 & \text{if }SD_{i}\leq \frac{1}{M}\sum_{k\in \{brain\}}SD_{k}\\ 0 & \text{if }SD_{i}>\frac{1}{M}\sum_{k\in \{brain\}}SD_{k} \end{array},$$ (6)

gauges the proportion of white matter voxels with lower strength in SD than the whole-brain average and was used to measure the true negative rate of SD. The gray and white matter parcelations provided with the Q1 release of the HCP dataset were used for these purposes; and

$$Reproducibility=1-\frac{1}{M}\sum_{i\;\in \;brain}^{M}abs\left(\frac{{SD}_{i}^{REST1}-{SD}_{i}^{REST2}}{{SD}_{i}^{REST1}+{SD}_{i}^{REST2}}\right).$$ (7)

### Reliability

The test-retest reliability of SD were evaluated for each imaging voxel using two-way mixed single measures intraclass correlation [38],

$$ICC(3,1)=\frac{BMS-EMS}{BMS+(k-1)EMS},$$ (8)

Specifically, each subject’s measurement (SD at each voxel) is assumed to be a random sample from a population of measurements. Case 3 (fixed effects) was selected because the measures were obtained in two different sessions (*k* = 2, REST1 and at REST2; “the raters”), which are the only sessions of interest. Previous test-retest reliability studies on FC have also used Case 3. In this work ICC was based on single measurements ICC(3,1), in order to be consistent with previous test-retest reliability studies. ICC(3,1) was mapped in the brain in terms of between-subjects (BMS) and residuals (EMS) mean square values computed for each voxel using the IPN matlab toolbox (http://www.mathworks.com/matlabcentral/fileexchange/22122-ipn-tools-for-test-retest-reliability-analysis) and the SD maps corresponding to REST1 and REST2 sessions (*k* = 2) from all subjects (*L* = 40):

$$\begin{array}{ll} BMS & =\frac{1}{L-1}{\sum_{j=1}^{L}\left(\sum_{i=1}^{k}SD_{ij}-\frac{1}{L}\sum_{j=1}^{L}\sum_{i=1}^{k}SD_{ij}\right)}^{2}\\ EMS & =\frac{1}{\left(L-1\right)\left(k-1\right)}\sum_{i=1}^{k}\left[{\sum_{j=1}^{L}{\left(SD_{ij}-\frac{1}{k}\sum_{i=1}^{k}SD_{ij}\right)}^{2}-\left(\sum_{j=1}^{L}SD_{ij}-\frac{1}{k}\sum_{j=1}^{L}\sum_{i=1}^{k}SD_{ij}\right)}^{2}\right] \end{array}.$$ (9)

Note that ICC(3, 1) coefficients range from 0 (no reliability) to 1 (perfect reliability).

### Between subject variability in SD and gray matter

We ran an additional analysis to assess the contribution of common and uncommon gray matter voxels to the individual differences in SD. Two gray matter masks were generated for each subject pair, one showing common gray matter voxels (GM) between the two subjects and one showing the combination of uncommon gray matter voxels (No GM) that are exclusive to each subject. Two maps were computed, MBSD^GM^ and MBSD^No GM^, reflecting the average between-subject differences in SD within each of these masks.

### Randomness and entropy

The IDL algorithm KSTWO [39], which computes the Kolmogorov-Smirnov statistic and associated probability that two arrays are drawn from the same statistical distribution, was used to compare the distribution of the time-varying *l*FCD against that of a uniform random variable. The time-varying *l*FCD datasets from the 2 sessions and 2 phase encoding directions were concatenated to form 4D datasets with 92 time points and to increase statistical power. The non-parametric Kolmogorov-Smirnov test was computed as a function of time for each voxel in the brain. The probability that the *l*FCD data have random distribution was computed for each subject and then averaged across subjects.

Shannon entropy was used to assess the amount of information in the time-varying *l*FCD. Specifically, for each subject the 4 rfMRI time series were concatenated into a single time series with 92 time points and entropy maps were computed according to

$$H_{2}=-\sum_{i=1}^{n}p_{i}{\text{log}}_{2}p_{i},$$

where *p*_i_ is the probability of *l*FCD = *i*.

### Statistical methods

A full factorial design was used to compare metrics from different sessions (REST1 vs REST2), phase encoding directions (LR vs RL), correlation thresholds (R < 0.3, 0.4 or 0.5), sliding-window shapes (rectangular vs hamming) and length (100 time points vs 200 time points), or GSN (ON vs OFF). The statistical parametric mapping package (SPM8) was used for this purpose. Statistical significance was set by a P_FWE_ < 0.05, corrected for multiple comparisons at the cluster level with the random field theory and a family-wise error correction with a cluster-forming threshold of P < 0.001 and a minimum cluster size of 200 voxels.

## Results

### *l*FCD

**Fig 2:**
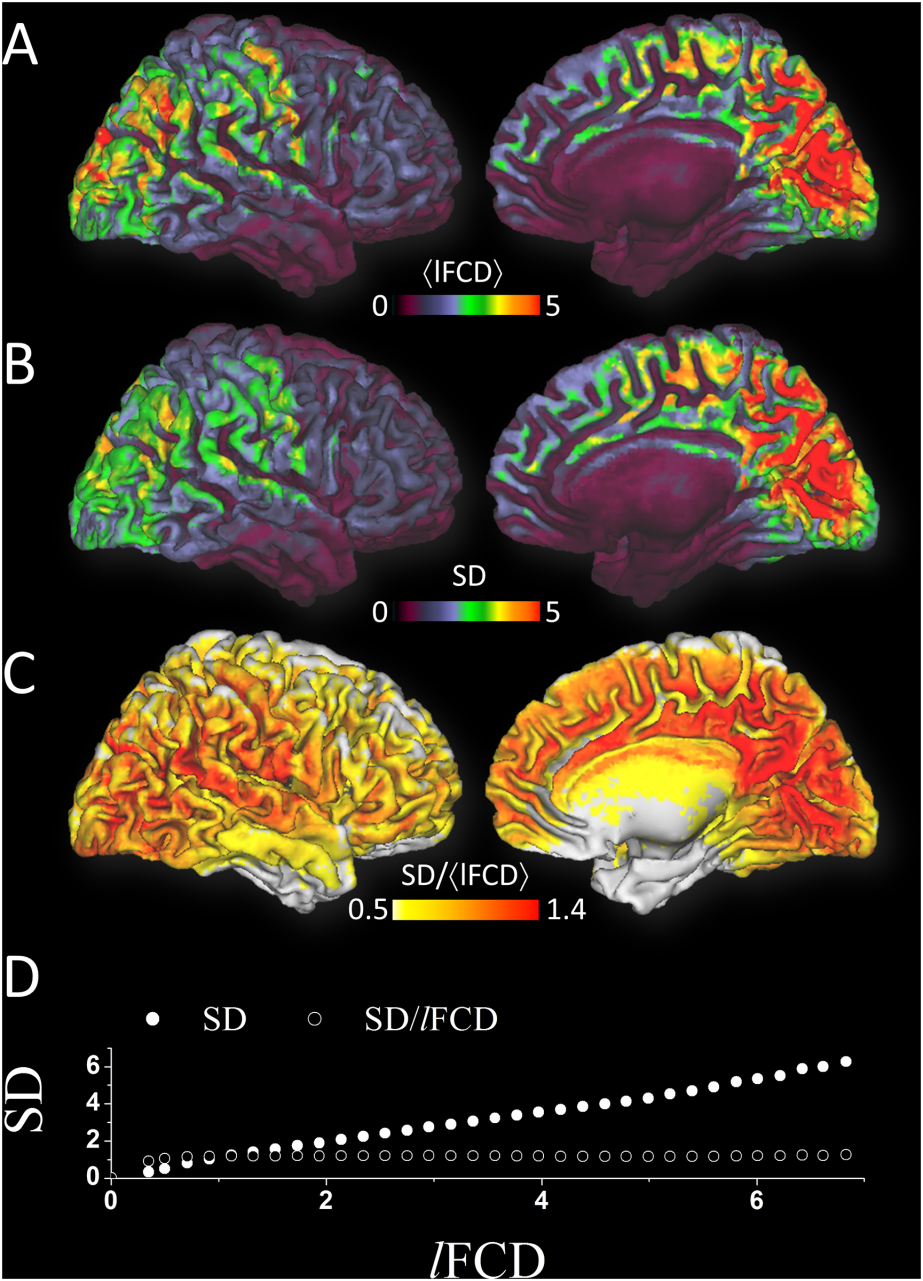
Temporal variability at the group level. Average distributions of strength (*l*FCD; A) and standard deviation (SD; B) of the *l*FCD as well as their ratio (C) across subjects showing brain areas where these metrics had higher values than twice (A and B) their whole brain averages, superimposed on axial (right), sagittal (middle) and coronal (left) views of the cortical and subcortical gray matter template developed using the HCP structural scans (pipeline 4). *l*FCD, SD and SD/*l*FCD ratio maps were computed for each subject and averaged. Note that voxels in white matter and cerebrospinal fluid were not excluded and that the imaging threshold (twice the whole brain average) was the only criterion used to display the patterns. The scatter plot (D) demonstrates the lack of association between the relative temporal dynamics (SD/*l*FCD) and the strength of the *l*FCD hubs. Image voxels were sorted by the strength of the rescaled *l*FCD and averaged into bins of *l*FCD = 0.1, independently for *l*FCD, SD and for the SD-to-*l*FCD ratio.

In most cortical regions the average *l*FCD across subjects was stronger than the whole brain mean *l*FCD (Fig 2A). The *l*FCD patterns predominantly followed the shape of cortical gray matter, and had minimal overlap with white matter and cerebrospinal fluid. However, inferior ventral, orbitofrontal, anterior temporal and insular cortices and subcortical regions showed attenuated *l*FCD, which might reflect the lower sensitivity of the 32-channels head coil in deep brain regions [40].

### Time-varying *l*FCD

**Fig 3:**
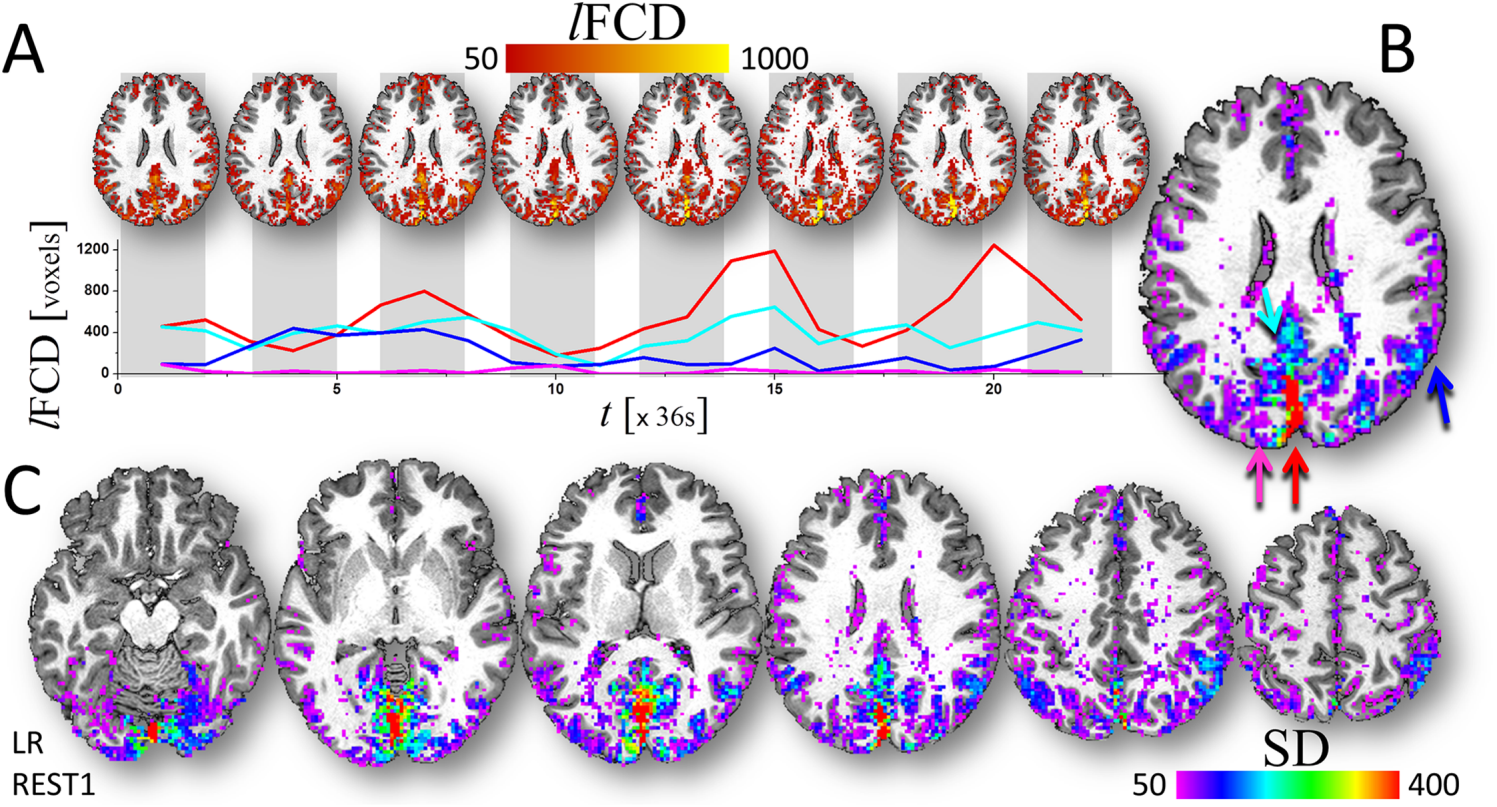
Temporal variability at the individual level. (A) Exemplary series of dynamic *l*FCD maps (in voxels; i.e. without grand mean global scaling) from a typical resting state HCP dataset superimposed on an axial view of the T1 weighted brain structure (top) and *l*FCD time courses (colored lines) corresponding to four different voxels from gray matter regions (colored arrows). The standard deviation (SD; in voxels) maps in B and C quantify the temporal dynamics of the *l*FCD metric in the brain for a single individual. Pipeline 4.

The sliding-window analysis demonstrated that *l*FCD patterns varied as a function of time, despite the high statistical significance of the static *l*FCD in the whole brain (Fig A in S1 File). The single-subject data in Fig 3A exemplifies the dynamics of the *l*FCD over the course of the 14 min resting-state scanning and shows that *l*FCD time courses varied from region to region. We used the temporal standard deviation, SD, to map the temporal variability of the *l*FCD (Fig 3). Across subjects, the variability of the *l*FCD as a function of time was high in medial occipital and parietal cortices, angular gyrus, superior and inferior parietal cortex, and medial prefrontal cortex, which are regions with high static *l*FCD. Specifically, in these regions SD was two times higher than the whole brain mean (Fig 2B) and similar to the distribution of the static *l*FCD (Fig 2A). The remarkable gray matter sensitivity, reproducibility and specificity of the SD (Fig B in S1 File) were used as benchmark criteria to assess the effect of image preprocessing steps on the dynamics of the *l*FCD.

### Correlation threshold

The static (*l*FCD) and dynamic (SD) connectivity density patterns did not change significantly as a function of the correlation threshold used for FCDM in any brain region (P_FWE_ > 0.05; Fig C in S1 File).

### Global signal normalization

**Fig 4:**
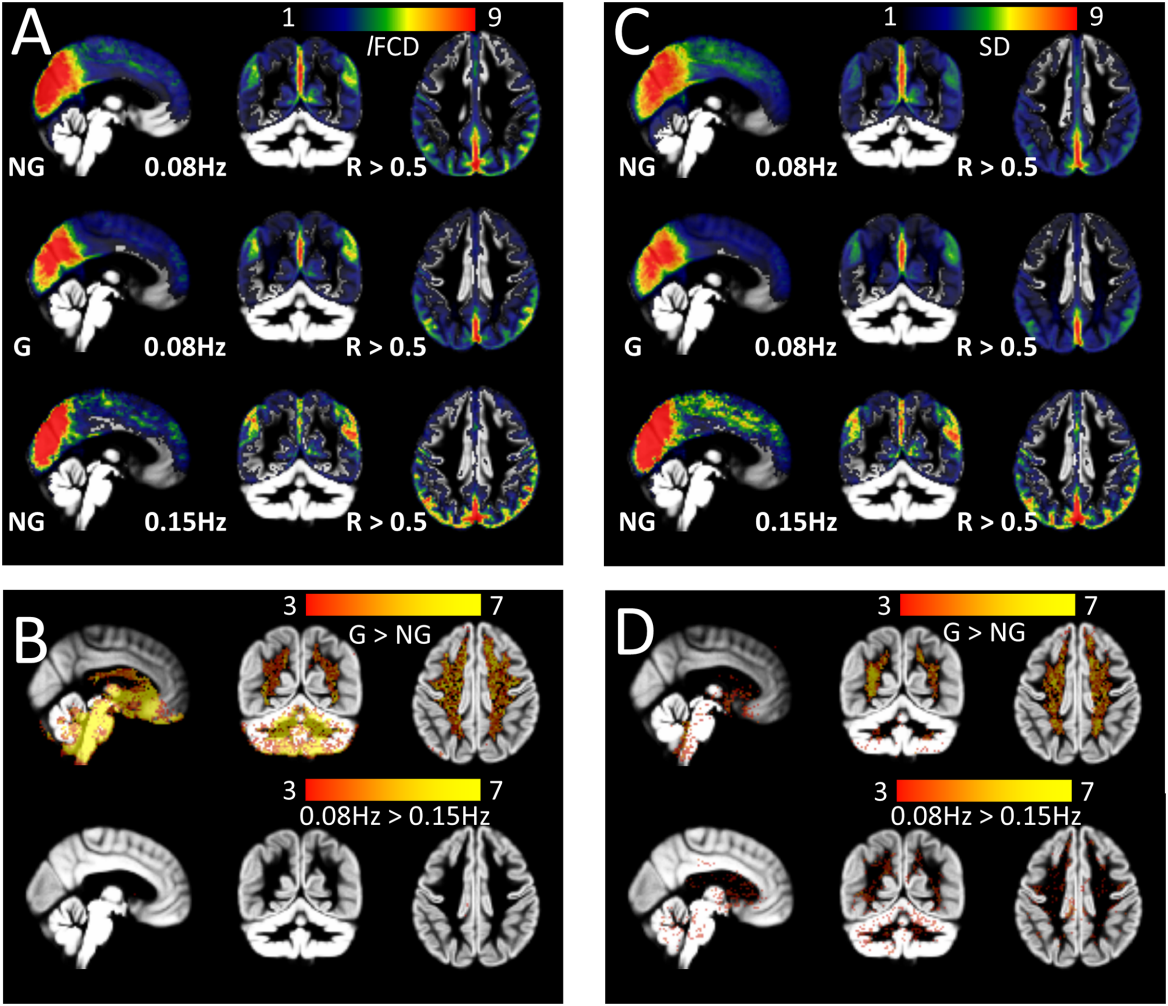
Effect of GSN. Average *l*FCD (A) and SD (C) maps across subjects with (G) and without (NG) GSN, with 0.08Hz or 0.15Hz low-pass filtering, and their statistical differences (two-sided t-score; B and D) superimposed on axial (right), sagittal (middle) and coronal (left) views of the cortical and subcortical gray matter template developed using the HCP structural scans.

GSN did not change static and dynamic connectivity density patterns in cortical gray matter regions (P_FWE_ > 0.05) but increased them in cerebrospinal fluid (CSF), white matter, subcortical gray matter and cerebellum (P_FWE_ < 0.05; Fig 4).

### Sliding-window length and shape and low pass filter

There were moderate changes in the SD due to window shape and size (Fig D in S1 File), yet these changes were not statistically significant in any brain region (P_FWE_ > 0.05; Fig D in S1 File). Similarly, there were no significant differences in SD or *l*FCD between 0.15Hz low-pass filtered datasets and 0.08Hz low-pass filtered datasets (Fig 4).

### SD-to-*l*FCD ratio

The spatial distribution of SD matched that of *l*FCD patterns (Fig 2A and 2B) suggesting that hub regions with high *l*FCD have prominent temporal dynamics. Thus the mean magnitudes of *l*FCD and SD across subjects were highly correlated across brain regions (Fig 2). The SD-to-*l*FCD ratio was used to normalize SD measures by the amplitude of the *l*FCD independently for each subject (Fig 2C). In order to assess the relationship between *l*FCD and SD, image voxels were sorted by the strength of the rescaled *l*FCD and averaged into bins of *l*FCD = 0.1, independently for *l*FCD, SD and for the SD-to-*l*FCD ratio (Fig 2D). Whereas SD and *l*FCD showed strong linear association across voxels, the SD-to-*l*FCD ratio did not show significant linear effects with *l*FCD (Fig 2D), regardless of GSN conditions and correlation thresholds used to compute the dynamic *l*FCD patterns (Fig E in S1 File). Note that the SD-to-*l*FCD ratio (coefficient of variation) should capture connectivity that was highly variable due to factors other than hubness.

### *l*FCD vs ALFF

**Fig 5:**
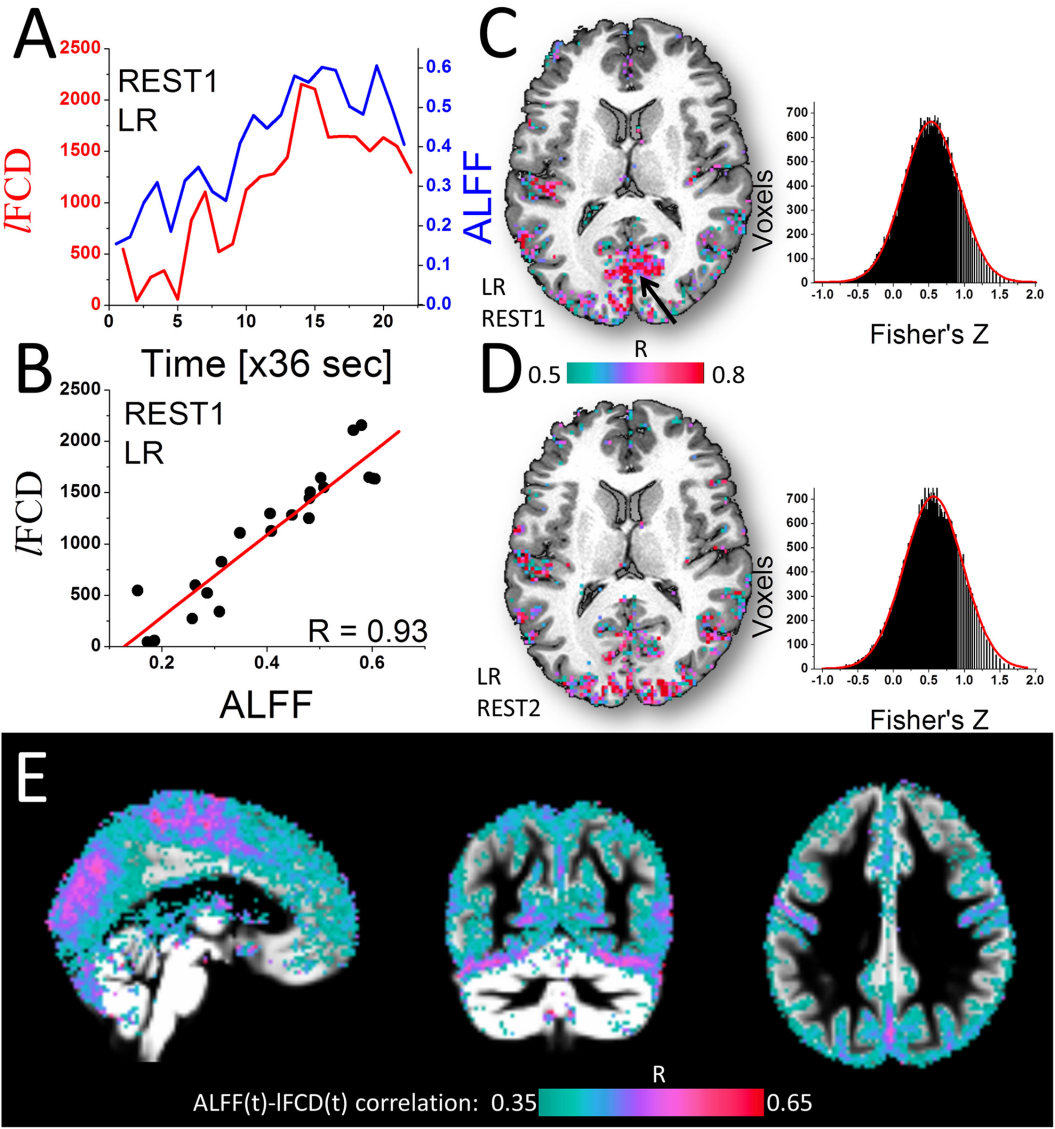
Dynamic association between *l*FCD and ALFF. Typical single subject data (A-D) showing (A) exemplary time courses from a voxel in primary visual cortex (black arrow in C) for *l*FCD (red) and ALFF (blue) and their correlation as a function of time (B), as well as the spatial distribution of their temporal correlation coefficients in the brain (C and D), which after Fisher’s transformation (histograms) had normal distribution (red Gaussian curve fits). (E) Distribution of the average ALFF-*l*FCD correlation coefficients across subjects superimposed on three orthogonal views of the gray matter template.

The sliding-window analysis demonstrated a linear association between *l*FCD and the amplitude of the spontaneous fluctuations in the brain. Specifically, brain regions with higher *l*FCD than the whole brain average exhibited time-varying *l*FCD patterns that were synchronous with time-varying ALFF patterns (Fig 5). The Pearson correlation used to map the linear association between *l*FCD and ALFF (Fig 5B) revealed correlation patterns that overlapped with the cortical gray matter regions housing the most prominent *l*FCD hubs in each individual (Fig 3C). These temporal correlation patterns had normal distribution across voxels and were highly reproducible across sessions (Fig 5C and 5D) and phase encoding directions (not shown). The average *l*FCD-ALFF temporal correlation maps across subjects revealed a strong linear association between time-varying patterns of *l*FCD and ALFF (Fig 5E). Specifically, insula as well as occipital, posterior and superior parietal and dorsolateral prefrontal cortices and premotor areas showed high temporal correlation between the time-varying *l*FCD and ALFF metrics.

### Effects of head motion

**Fig 6:**
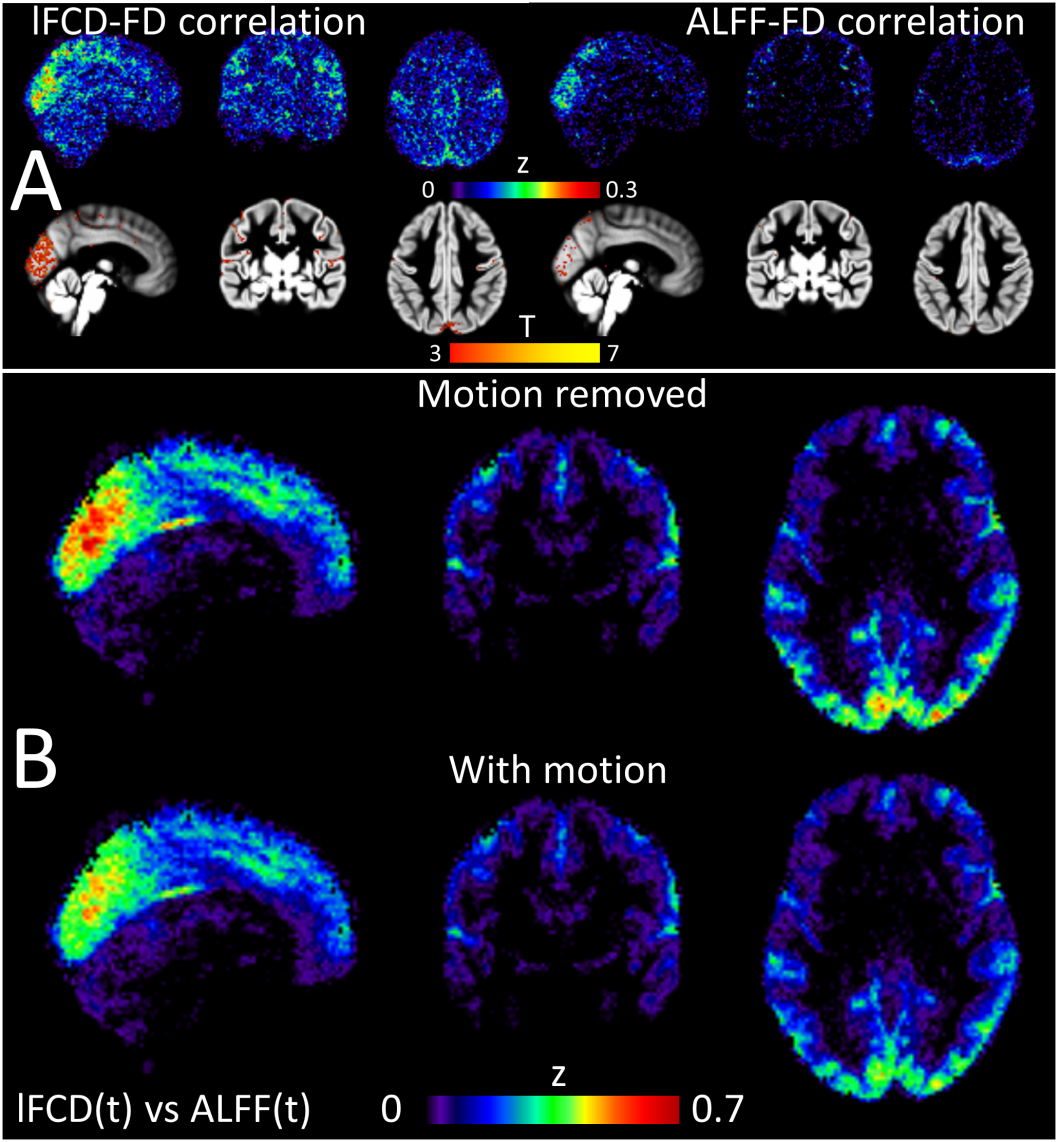
Effects of head motion on FC dynamics. (A) Mean Fisher’s z-score values (top row) and their statistical significance across subjects superimposed on three orthogonal views of a gray matter template (t-test; bottom row), demonstrating the linear correlation between framewise displacements (FD) and the FC metrics (*l*FCD and ALFF) in the brain as a function of time. (B) Three orthogonal views showing the distribution in the brain of the group mean Fisher’s z-scores from partial correlation analyses (“Motion removed”; top row) and from standard Pearson correlation analyses (“With motion”; bottom row).

The average head motion during the 14 minutes long resting-state scans did not differ between MRI sessions or phase encoding directions across subjects (P > 0.2, paired t-test; 〈FD〉 = 0.176 ± 0.05 mm). The 72s-rectangular sliding-window analysis revealed that the time-varying FD had a temporal standard deviation of 0.02 ± 0.01 mm, which did not differ between sessions or phase encoding directions (P > 0.71) and was smaller than the standard deviation of the static FD (i.e., average FD within a session) across subjects (P < 0.001). Pearson correlation was used to assess the linear association of FC and *l*FCD or ALFF patterns as a function of time for each subject. A t-test of the Fisher’s z-transformed correlation maps demonstrated that increases in FD slightly increased *l*FCD in occipital, temporal motor and premotor cortices and insula, and ALFF in the occipital cortex (P_FWE_ < 0.05; Fig 6A). The partial correlations used to remove the effect of head motion in the linear association between time-varying patterns of *l*FCD and ALFF showed that head motion effects on the association between *l*FCD and ALFF were not statistically significant (P > 0.05, uncorrected; Fig 6B).

### Gray matter sensitivity

**Fig 7:**
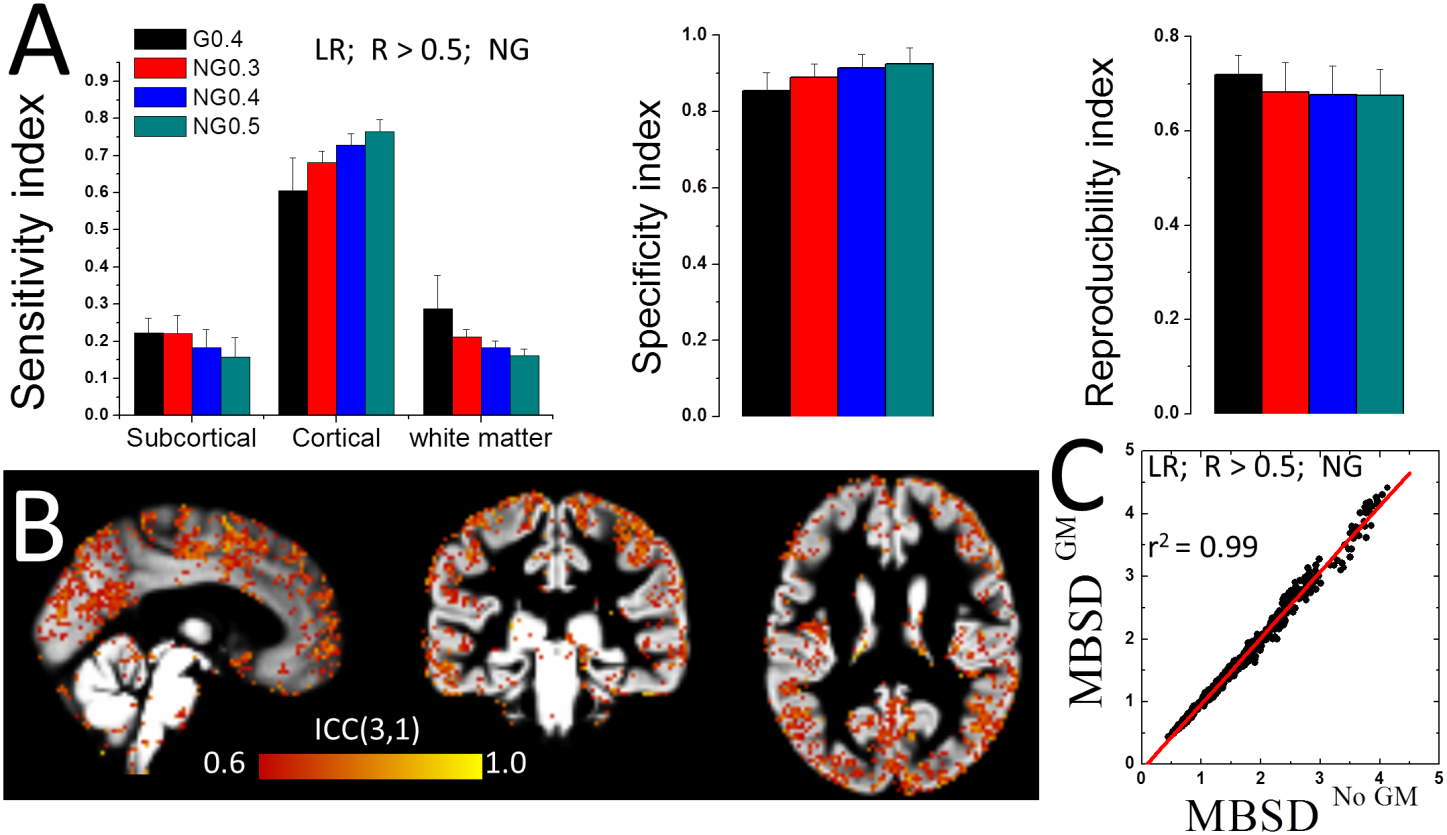
*l*FCD dynamics: Gray matter specificity and test-retest reliability. (A) Average sensitivity index for SD in subcortical and cortical gray matter and white matter, and average reproducibility and specificity indices across subjects for each of the processing pipelines in Fig 1. (B) Two-way mixed single measures intraclass correlation ICC(3,1) maps at 2-mm isotropic resolution depicting regional variability in test-retest reliability for SD (pipeline 4). (C) Scatter plot showing the linear association of the mean between-subject SD-differences (MBSD) across voxels in overlapping (No GM) and non-overlapping (GM) gray matter for all potential pairs of subjects (pipeline 4). Error bars are standard deviations.

The *sensitivity* index, which captures the proportion of SD within a tissue of interest, was higher for cortical grey matter than for white matter and subcortical gray matter (including cerebellum). The sensitivity index in cortical gray matter reached maximal value (85 ± 3%) for SD maps computed without GSN and using a correlation threshold *of* 0.5 (Fig 7A). The use of GSN or lower correlation thresholds significantly reduced the SD gray matter sensitivity index (P < 10^−9^; paired t-test, df = 39). The SD sensitivity index did not differ between LR and RL runs or between the REST1 and REST2 sessions.

### Reproducibility

The *l*FCD-SD patterns computed without GSN had *reproducibility index* of 67 ± 6% across sessions which did not significantly differ as a function of correlation thresholds (Fig 7A). GSN, improved the reproducibility of the SD maps to 72 ± 4% (P < 10^−13^; paired t-test, df = 39). Voxelwise analysis contrasting REST1 and REST2 did not show statistically significant SD differences between sessions in any brain region.

### Specificity

The *specificity index* of the *l*FCD-SD patterns was better than 85% across threshold and GSN conditions (Fig 7A), sessions and phase encoding directions (not shown). The specificity index gradually improved with higher correlation thresholds and with the lack of use of GSN (P < 10^−10^, paired t-test, df = 39).

### Reliability

Intraclass correlation analyses of test-retest datasets demonstrated the high reliability (ICC(3,1) > 0.5) of the SD in cortical regions (Fig 7B). Whole-brain test-retest reliability of SD slightly improved with higher correlation threshold (ICC(3,1) ^NG^_R>0.5_—ICC(3,1) ^NG^_R>0.3_ = 0.009 ± 0.0004, mean ± SE) and with GSN (ICC(3,1) ^G^_R>0.4_—ICC(3,1) ^NG^_R>0.4_ = 0.045 ± 0.0006).

### SD variability in relation to gray matter

**Fig 8:**
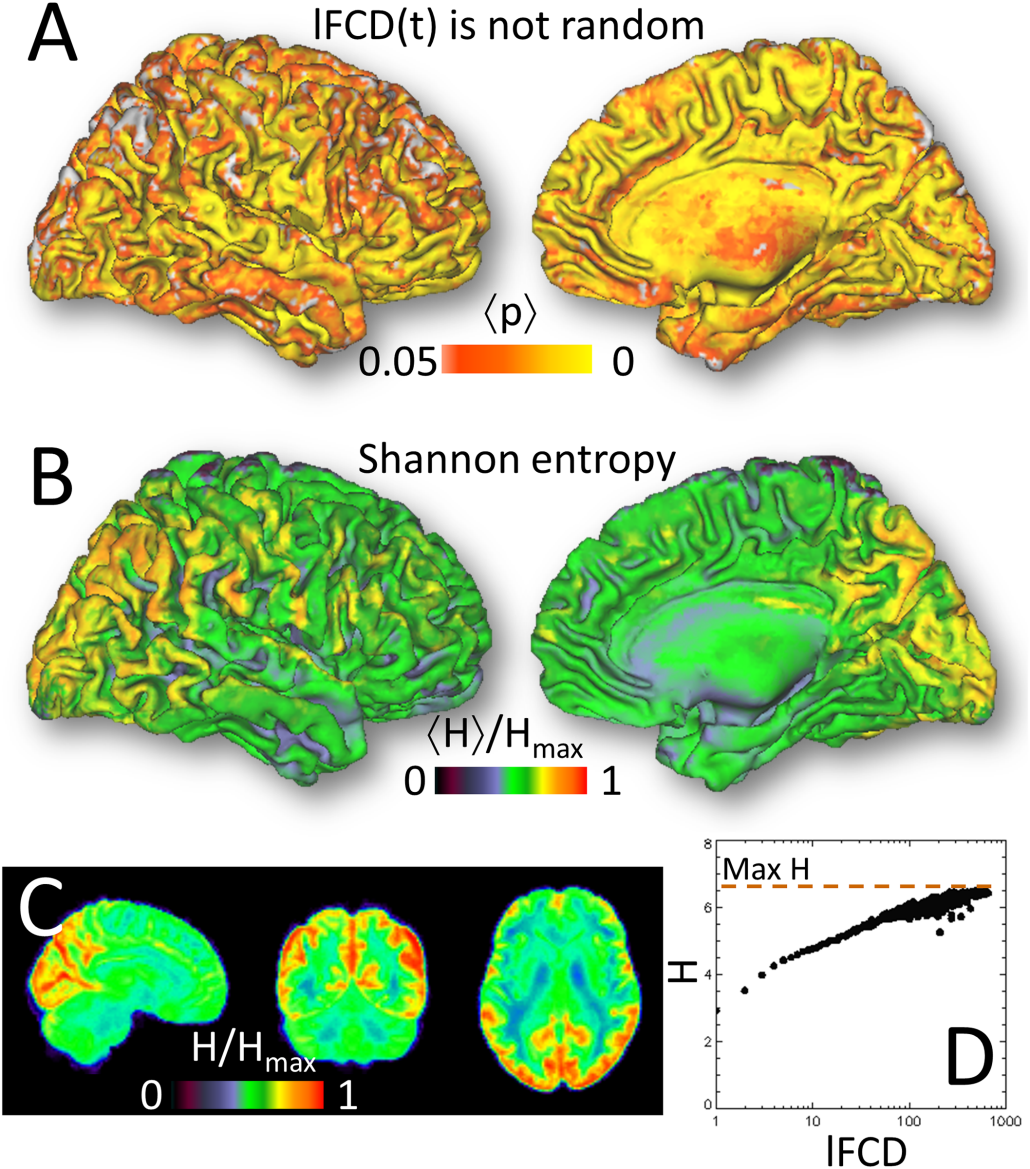
Randomness and Entropy. Statistical maps from Kolmogorov-Smirnov test (see methods) averaged across subjects, highlighting brain regions where the cumulative distribution function of the time-varying *l*FCD differed from that of a uniform random variable (**A**). Average entropy across subjects superimposed on **B**) lateral and medial views of the brain surface and **C**) three orthogonal views of the brain. The color bar displays the average entropy, H, across subjects relative to the maximal entropy, H_max_ = log_2_(92), effectively attained with the concatenation of 4 time series (4 × 23 temporal windows) from each subject. **D**) Exponential saturation of H with *l*FCD in typical subject data.

Since the folding patterns of cortical gray matter are highly variable across individuals [41] we assessed the inter-individual *l*FCD differences in relation to the folding patterns of cortical gray matter for all pairs of subjects. Cortical gray matter and SD patterns overlapped for each individual but varied across individuals. On average across all potential pairs of subjects, the cortical gray matter patterns of two randomly selected subjects overlapped in 55600 ± 2000 voxels (mean ± standard deviation) in which the MBSD^GM^ in SD was 1.644 ± 0.815 times higher than the whole brain mean (NG, R> 0.5). For voxels which did not overlap in cortical gray matter for the two subjects (55500 ± 1800 voxels) the MBSD^No GM^ in SD was 1.646 ± 0.767 times higher than the whole brain mean. The MBSD measures within overlapping and non-overlapping gray matter areas showed significant correlations across subjects (r^2^ = 99, Fig 8C). This observation was not affected by different *l*FCD-thresholds, GSN conditions or phase encoding directions (Fig G in S1 File). Thus, inter-individual differences in temporal variability of *l*FCD were not significantly associated to inter-individual differences in the spatial distribution of cortical gray matter.

### Randomness and entropy

In most brain regions the cumulative distribution function of the time-varying *l*FCD differed from that of the random variable (Fig 8A). Brain regions with increased static *l*FCD also demonstrated increased entropy (Fig 8B–8D).

## Discussion

Here we assess for the first time the temporal dynamics of the *l*FCD, a graph theory metric of functional organization in the human brain. Specifically, using a sliding-window approach in resting-state fMRI data collected with high spatiotemporal resolution (2-mm isotropic; 0.72 s), we reveal pronounced temporal variability in *l*FCD in visual and posterior parietal cortices encompassing the default-mode network regions. The temporal variability in *l*FCD was restricted to gray matter and temporal changes in *l*FCD were associated to temporal changes in ALFF.

The present study provides evidence that the temporal dynamics of the *l*FCD is synchronous with the temporal dynamics of ALFF. This dynamic association, which predominantly occurred in medial occipitoparietal regions, is consistent with the temporal dynamics observed for the connectivity of posteromedial cortex seeds [42,43]. Different from previous studies that reported temporal variability in the strength of the functional connectivity of large ROIs in the posteromedial cortex (a 12-mm diameter sphere located in ventral precuneus [43] or four 6-mm diameter spheres located in dorsal precuneus [42]), the present study quantifies temporal variability of functional connectivity at 2-mm isotropic resolution. In addition, we document for the first time gray matter sensitivity and specificity, and the reliability of the temporal variability of the hubs.

The strength and the temporal variability of the *l*FCD as well as its association with ALFF were maximal in occipital and medial parietal regions. These brain regions have the highest metabolism in brain [44,45], which presumably supports the energy requirements of higher communication rate in these regions [46]. The probability distribution of time-varying *l*FCD could not be explained by randomness using the Kolmogorov-Smirnov test. Shannon entropy also suggests that processes other than random processes play a role in the temporal variability of *l*FCD. Specifically, we found that in posterior and medial occipitoparietal networks the *l*FCD had high entropy which increased in proportion to the strength of the hubs (Fig 8), suggesting high average flow of information in these regions.

*l*FCD gauges the strength of the local functionally connectivity hubs, regions with high degree, which are energy demanding [46] and target of neuropsychiatric disorders with disrupted energetics for which there is evidence of impaired energy generation (ie mitochondrial dysfucnction) as has been shown for Alzheimer’s, autism, schizophrenia alcoholism, attention-deficit hyperactivity disorder and aging energetics [17,19,47–54]. We assessed the temporal variability of the hubs using the sliding-window approach and quantified the temporal changes in the strength of the hubs using the temporal SD of the *l*FCD, similar to previous studies that quantified the temporal variability of the seed-voxel correlation patterns [15]. This work demonstrates for the first time that cortical *l*FCD hubs, which are densely located in occipitoparietal cortices, show significant temporal variability.

Similar dynamic changes have been observed in previous studies using seed-voxel correlations or ICA that reported temporal dynamics in functional connectivity metrics in lateral parietal and cingulate cortices and in the default-mode network [5,14,15] at significantly lower spatial resolution. The sharper connectivity patterns in the present study reflect the higher spatial resolution and faster sampling rate of the HCP datasets that resulted in *l*FCD patterns with greater sensitivity in relation to showing gray matter specific effects at the individual and group levels (preserved by the lack of spatial smoothing) and with reduced physiologic noise artifacts. Thus, the time-varying *l*FCD patterns were restricted to gray matter regions, regardless of the phase encoding direction used to collect the multiband echo-planar datasets as well as the processing approaches used to compute *l*FCD (e.g. correlation thresholds or GSN conditions).

This is the first study to quantify dynamic changes in functional connectivity capitalizing on the high spatial resolution of the HCP datasets. Specifically, the temporal variability (SD) in the strength of the *l*FCD hubs occurred in cortical gray matter with high sensitivity (up to 85 ± 3%) and high specificity (> 85%), and showed high reproducibility across sessions (up to 72 ± 4%). Furthermore, the SD patterns in cortical regions showed moderate to high reliability (ICC(3,1) > 0.5), suggesting lower within-subject than between-subjects variability. Whereas, for each individual the high *l*FCD-SD mapped onto cortical gray matter, the MBSD in SD was not sensitive to the amount of overlap in the gray matter across subjects, suggesting that the between-subject variability in the dynamics of *l*FCD does not reflect only between-subject variability in brain anatomy.

The temporal variability of the *l*FCD hubs was consistent with the proposed dynamic changes in network efficiency [1] and with results from previous studies that have documented lower functional connectivity and lower variability of the dynamic functional connectivity in patients with schizophrenia compared to control subjects [55]. In cortical gray matter the temporal variability of *l*FCD was proportional to the strength of the *l*FCD hubs (SD/*l*FCD ~1), suggesting that these regions are both highly connected and highly dynamic. This also suggests and that random processes may play a role in the temporal variability of the *l*FCD (random variables with higher mean are expected to have higher standard deviation) which might not be compatible with the existence of meaningful *l*FCD 'states’. At the cortical gray-white matter boundary, however, the strength of the hubs (i.e., amplitude of *l*FCD) did not fully account for the temporal variability in *l*FCD (SD/*l*FCD > 1). In these regions the *l*FCD was highly variable due to temporal noise, not purely due to hubness, suggesting that factors other than chance influenced the dynamics of *l*FCD in these regions.

A large SD may not necessarily reflect a highly dynamic signal (a random variable could have large SD but yet be classified as stationary). Using a non-parametric Kolmogorov-Smirnov test, we show that the time-varying *l*FCD is not a random variable in most brain regions, which supports the dynamic nature of these temporal fluctuations. We were not aware of a suitable statistical tool to test whether dynamic *l*FCD changes reflect the existence of ‘states’, and the time-varying *l*FCD in this study is not suitable for the detection of “*l*FCD states”. Our initial estimates suggest that detection of different “*l*FCD states” would require one order of magnitude longer scanning time (several hours) than those acquired under the HCP due to the high entropy of this dynamic metric. This study does not quantify the dynamics of *l*FCD in cerebellum and subcortical regions because the low sensitivity of the HCP datasets prevented *l*FCD quantification in deep brain regions, even at low correlation thresholds (R < 0.1; P > 0.16). In contrast to cortical regions, imaging deep brain regions do not benefit significantly from the use of multichannel coil arrays such as the 32-channel head coil used under the HCP. Furthermore, since the MRI signal is proportional to the voxel volume, a 66% signal-to-noise ratio (SNR) decrease is expected for 2-mm compared to 3-mm isotropic resolution. T1-relaxation also reduces SNR at high temporal resolution, because at TR = 0.72s and assuming T1 = 1s, the recovery of the longitudinal magnetization is 50%. Thus, white (random) rather than physiologic (nonrandom and proportional to SNR) noise could dominate the noise in cerebellum and subcortical regions, preventing observation of physiological signals in these regions at high spatiotemporal resolution. We were unable to quantify regional variations in T2-decay because the HCP datasets do not include T2 maps and were collected using single-echo multiband EPI. Since the time-varying *l*FCD findings in this study reflect BOLD signal fluctuations that are sensitive to T2-decay, part of the variability across regions could be attributed to differences in T2-decay.

To limit the effect of spurious connectivity fluctuations (false positives), our approach relied on a sliding-window technique with lengths of 72s and 144s, approximately the longest wavelength composing the BOLD signal [56,57]. Whereas Leonardi recommended choosing a window length exceeding the longest wavelength composing the BOLD signal, usually assumed to be ~100s, Zalesky showed that non-stationary fluctuations in functional connectivity can in theory be detected with much shorter window lengths (e.g. 40s), while maintaining nominal control of false positives. We did not observe significant differences in the dynamics of *l*FCD between sliding-windows of different lengths (72s versus 144s) or shapes (rectangular versus Hamming) (P_FWE_ > 0.05).

The data-driven nature of the proposed method allows quantification of dynamic properties of the functional connectivity and is fully compatible with voxelwise statistical approaches frequently used in neuroimaging. We also studied the effect of GSN on the dynamics of *l*FCD in gray matter because GSR is frequently used to control for scanner instabilities in resting-state functional connectivity. However global signal fluctuations could reflect true electrophysiological activity [58] and GSR may lead to spurious anti-correlations [59–61]. In this study, GSR increased reproducibility and reliability of SD in cortical gray matter, but spuriously increased SD in white matter and subcortical regions, which might confound results in these regions [62]. In addition the present study demonstrates the robustness of the SD to the correlation threshold and low-pass filter cut-off frequency used in the computation of *l*FCD. Besides neurophysiological factors, scanner noise and motion may lead to temporal variability in the *l*FCD index. While the present work does not dissociate the sources of temporal variability in *l*FCD in detail, future work may complement these findings by providing a more in-depth understanding of factors contributing to temporal changes in functional connectivity indices.

Overall, this work demonstrates temporal changes in data-driven functional connectivity metrics at high spatiotemporal resolution that are proportional to the strength of the connectivity hubs and the amplitude of the spontaneous MRI signal fluctuations in the brain. Whereas the biological origin of the dynamic connectivity changes remains unclear they occurred predominantly in gray matter, particularly occipitoparietal hubs, with high specificity, reproducibility and reliability.

## Supporting Information

S1 File Supporting statistical data analyses for “Temporal changes in local functional connectivity density reflect the temporal variability of the amplitude of low frequency fluctuations in gray matter”. (DOCX)
