# Supplementary material for: Temporal Changes in Local Functional Connectivity Density Reflect the Temporal Variability of the Amplitude of Low Frequency Fluctuations in Gray Matter
Source: PLoS One. 2016 Apr 26;11(4):e0154407. doi: 10.1371/journal.pone.0154407 (PMC4846007; doi:10.1371/journal.pone.0154407)
Supplement: S1 File — (DOCX) [file pone.0154407.s001.docx]

|  | Glossary of acronyms |
| --- | --- |
| ALFF | Amplitude of the low frequency fluctuations |
| BMS | Between-subjects mean square |
| BOLD | Blood oxygenation-level dependent |
| CSF | Cerebrospinal fluid |
| EMS | Error mean square |
| FC | Functional connectivity |
| FCDM | Fucntional connectivity density mapping |
| FD | Framewise displacement |
| fMRI | Functional magnetic resonance imaging |
| GM | Gray matter |
| GSN | Global signal normalization |
| H | Entropy |
| HCP | Human Connectome Project |
| ICA | Independent component analysis |
| ICC | Intraclass correlation coefficient |
| k | Number of sessions |
| L | Number of subjects |
| lFCD | Local functional connectivity density |
| M | Number of voxels |
| MBSD | Mean of between-subject differences |
| MNI | Montreal Neurological Institute |
| SD | Temporal standard deviation |
| SPM | Statistical parametric mapping |
| WM | White matter |

**
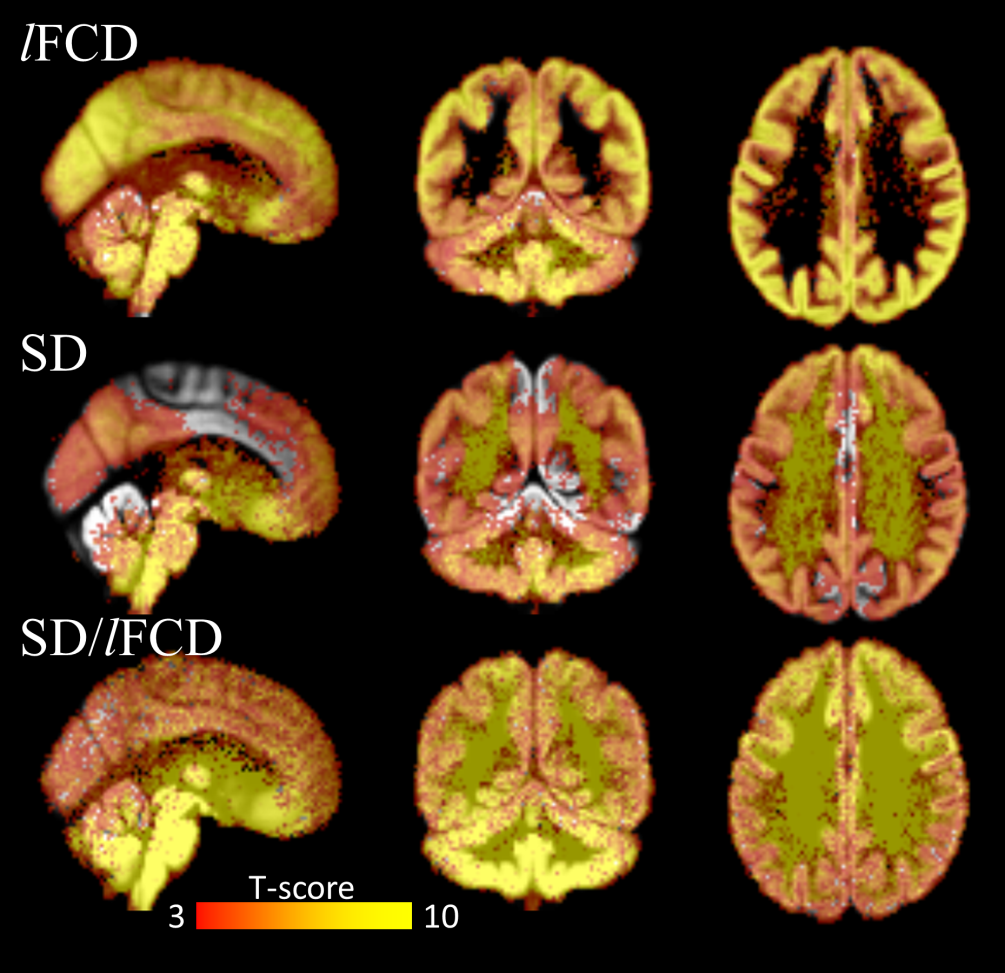
**

**Figure A: Statistical significance.** Statistical maps across subjects superimposed on axial (right), sagittal (middle) and coronal (left) views of the cortical and subcortical gray matter template developed using the HCP structural scans (pipeline 4).

**
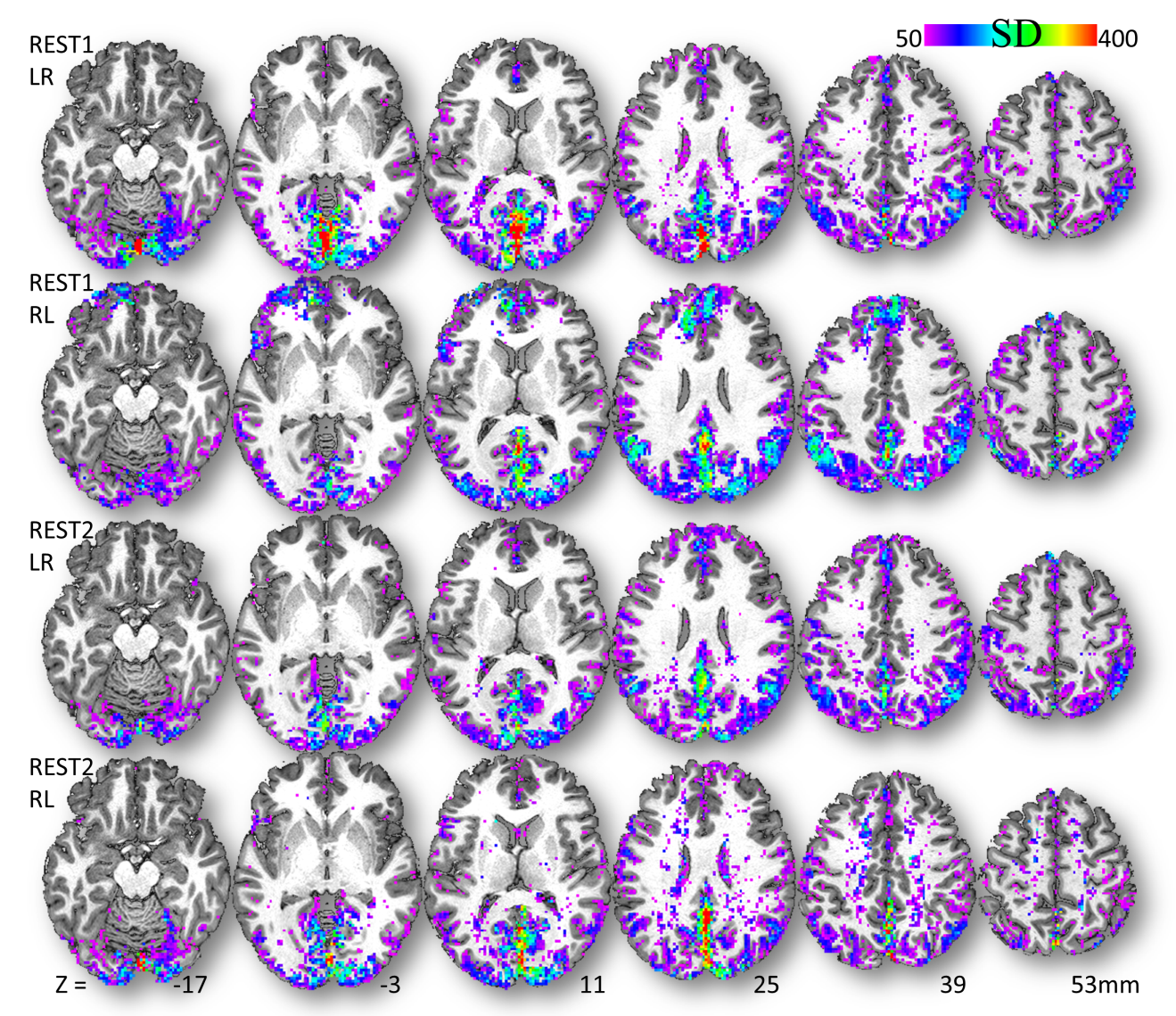
**

**Figure B: Reproducibility.** Exemplary standard deviation (SD) maps from a single individual for 2 different sessions (REST1 and REST2) and 2 phase encoding directions (LR and RL), superimposed on the corresponding brain structure. Pipeline 4

**
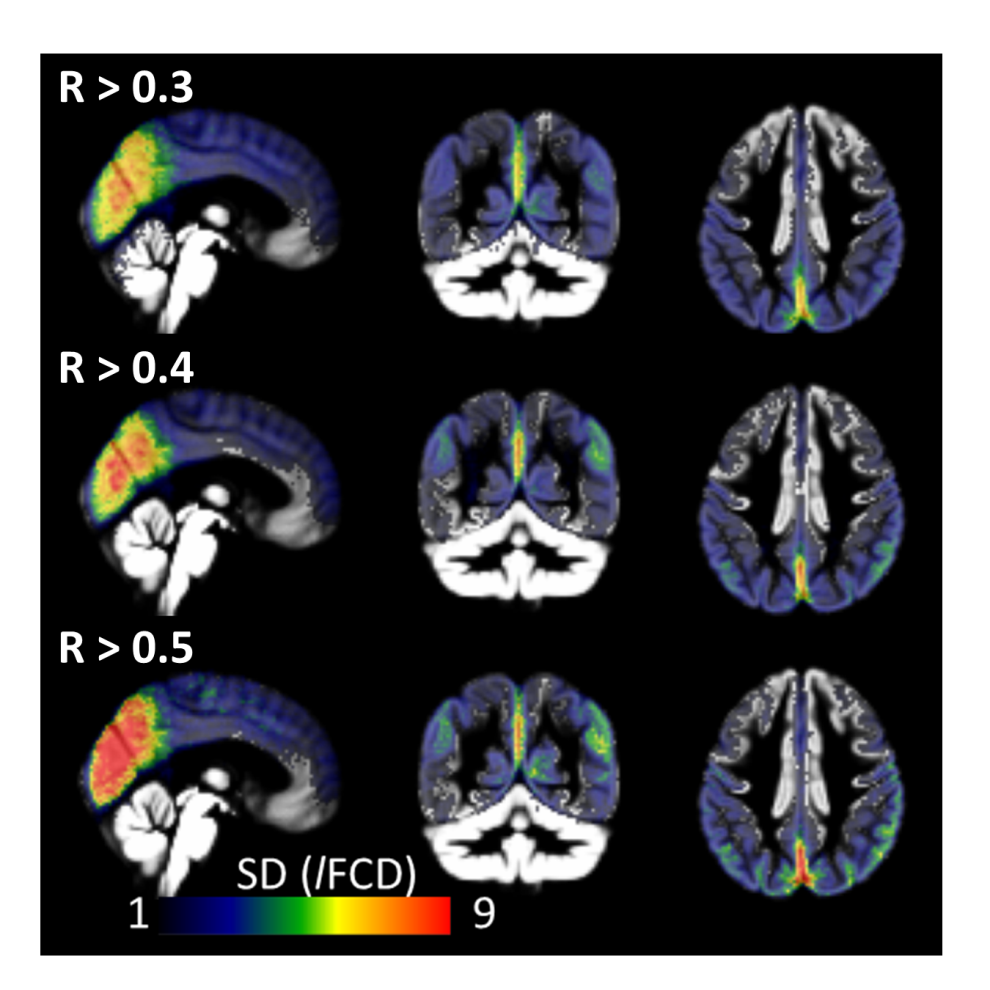
**

**Figure C: Effect of lFCD correlation threshold.** Average SD maps across subjects for different correlations thresholds (R) used in the computation of the lFCD, superimposed on axial (right), sagittal (middle) and coronal (left) views of the cortical and subcortical gray matter template developed using the HCP structural scans (pipeline 4).


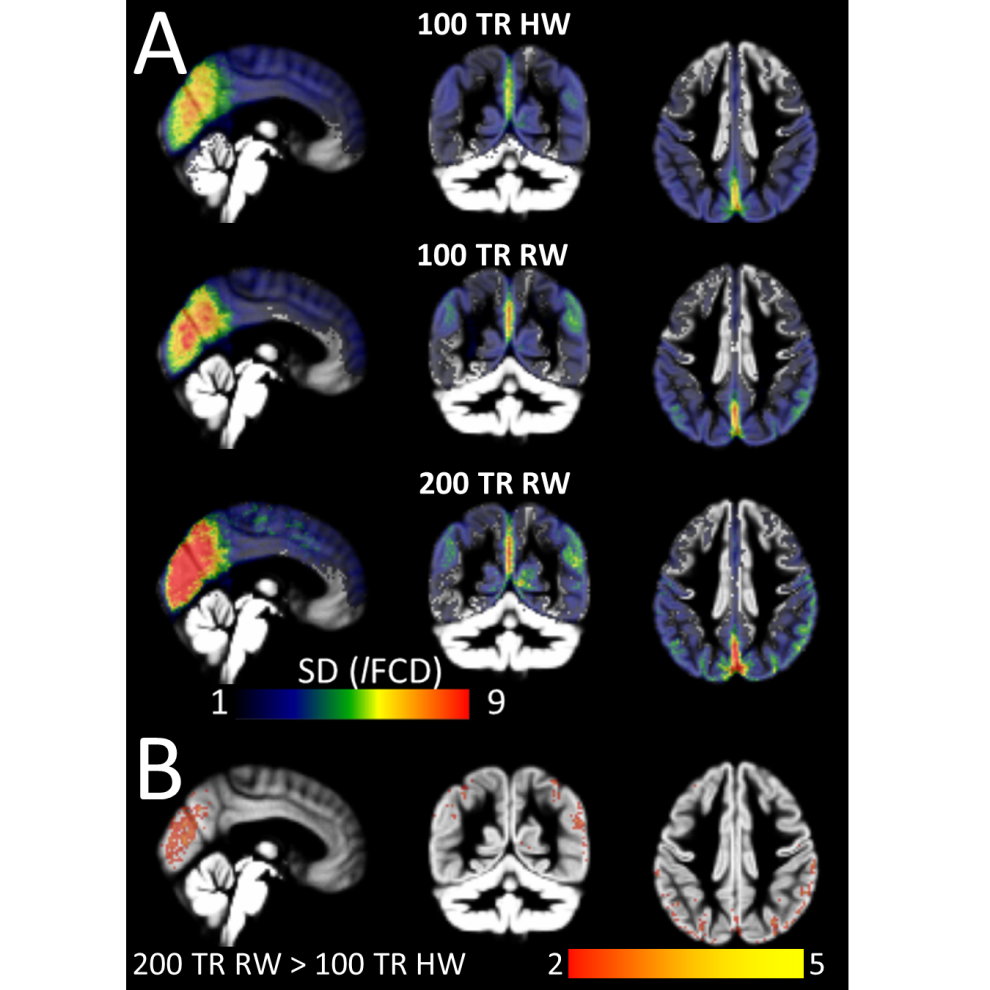


**Figure D: Effect of sliding window length and shape.** Average SD maps across subjects for rectangular (RW) and Hamming (HW) sliding windows of length 100 and 200 time points (A) and statistical significance of SD differences between the extreme conditions (B), superimposed on axial (right), sagittal (middle) and coronal (left) views of the cortical and subcortical gray matter template developed using the HCP structural scans.

**
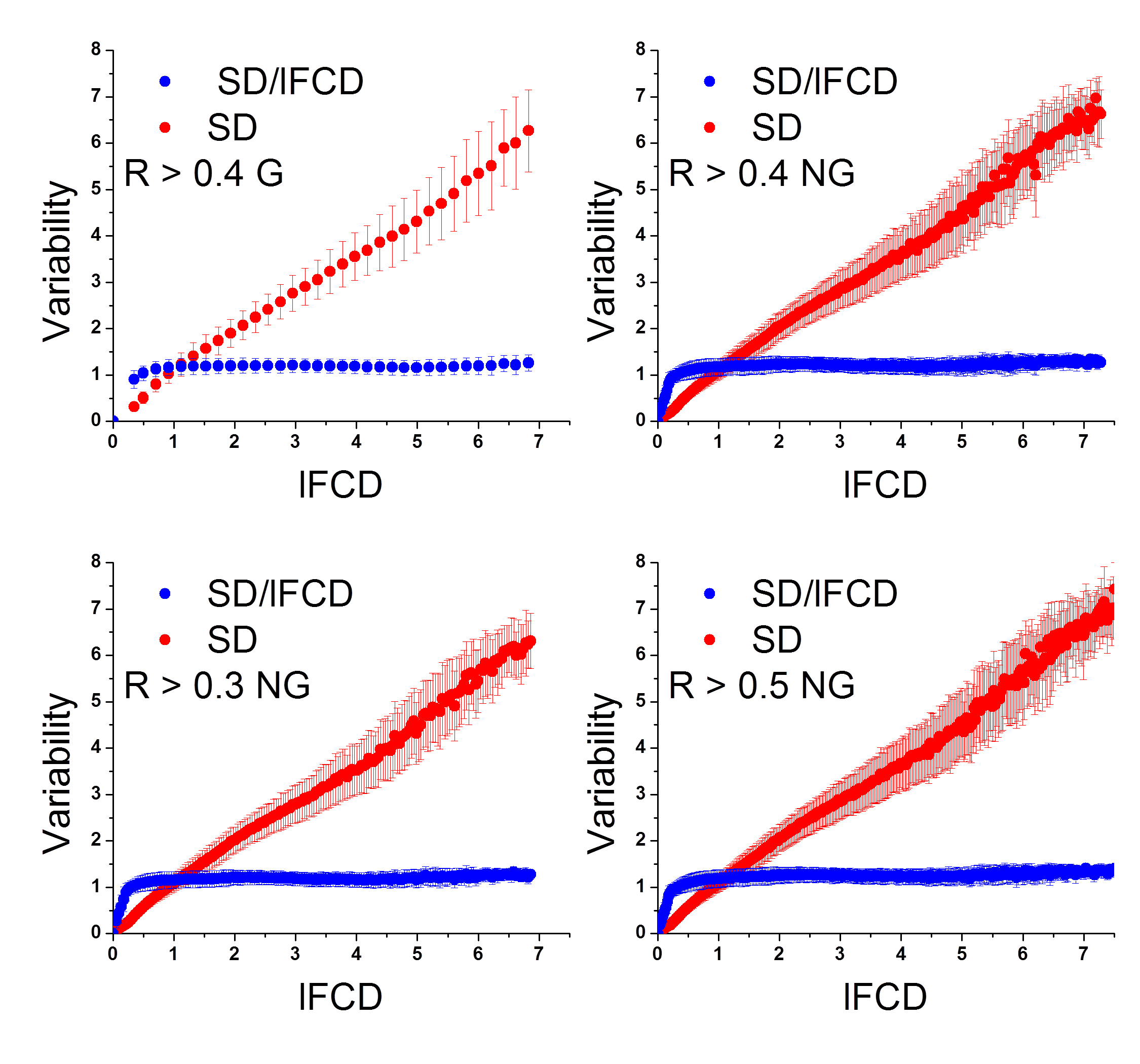
**

**Figure E.** Scatter plots showing the lack of association between the relative temporal dynamics (SD/*l*FCD) and the strength of the *l*FCD hubs (blue) for all pipelines.

**
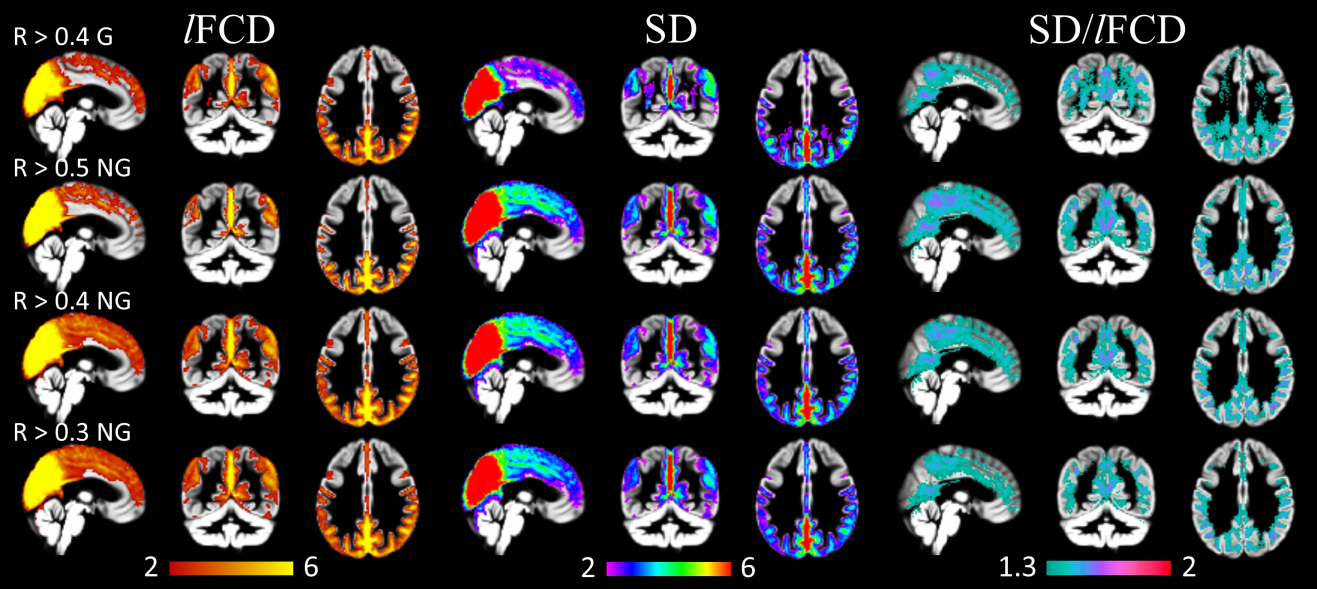
**

**Figure F.** Average distributions of strength (*l*FCD; left) and standard deviation (SD; middle) of the local degree as well as their ratio (right) across subjects showing brain areas where these metrics had higher values than twice (A and B) their whole brain averages for all pipelines, superimposed on three orthogonal views of the cortical and subcortical gray matter template.

**
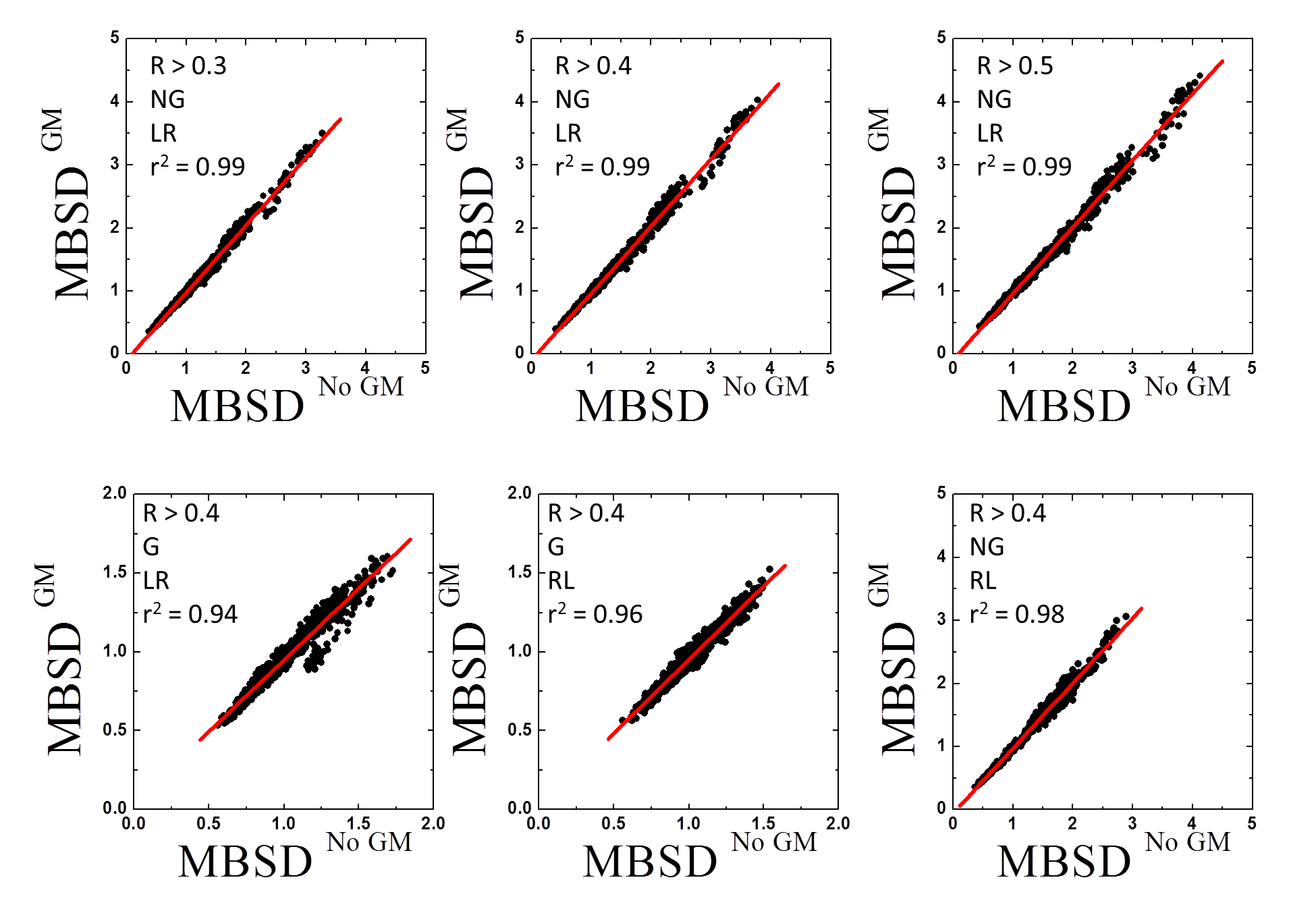
**

**Figure G:** Scatter plots showing the linear associations of the mean between-subject SD-differences (MBSD) across voxels in overlapping (No GM) and non-overlapping (GM) gray matter across subjects for all pipelines and phase encoding directions.


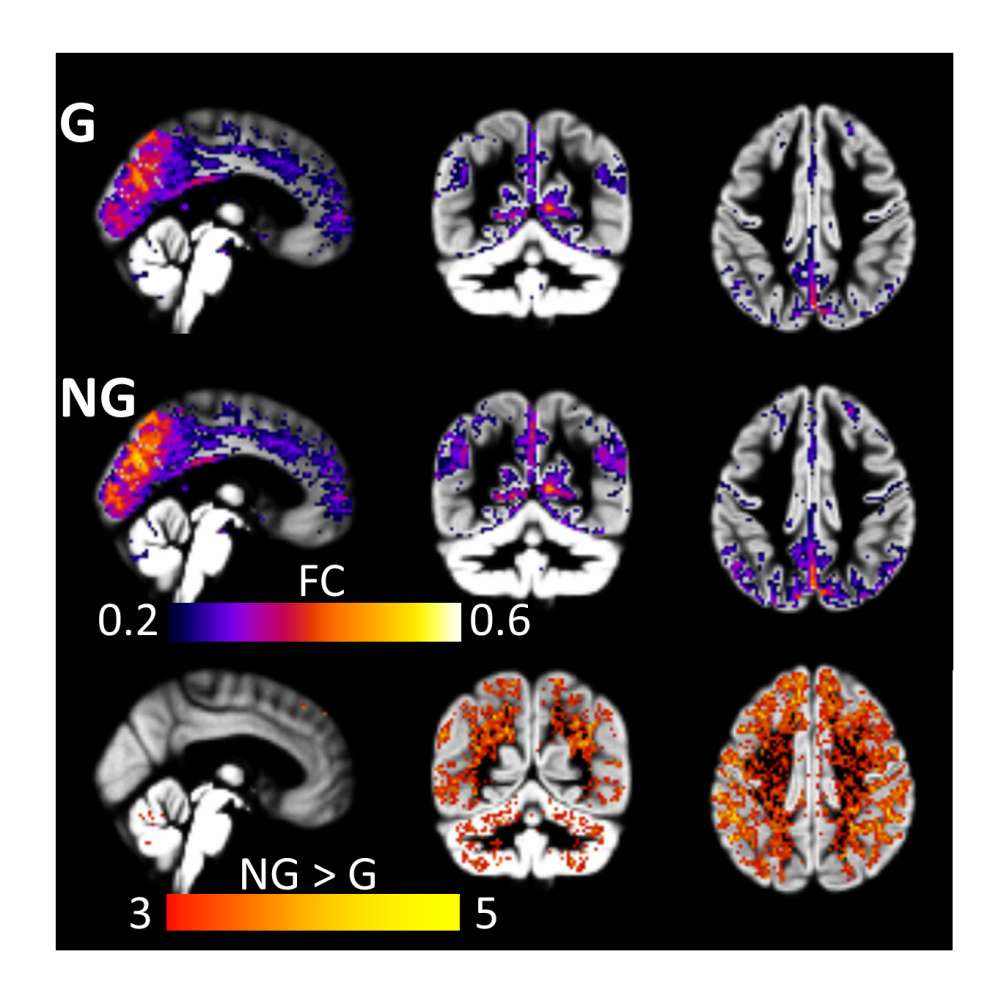


**Figure H**. Average FC maps across subjects with (G) and without (NG) global signal normalization and their statistical differences (t-score) superimposed on axial (right), sagittal (middle) and coronal (left) views of the cortical and subcortical gray matter template developed using the HCP structural scans.
